# Traumatic Brain Injuries: Pathophysiology and Potential Therapeutic Targets

**DOI:** 10.3389/fncel.2019.00528

**Published:** 2019-11-27

**Authors:** Si Yun Ng, Alan Yiu Wah Lee

**Affiliations:** ^1^Neurobiology/Ageing Program, Centre for Life Sciences, Department of Physiology, Yong Loo Lin School of Medicine, Life Sciences Institute, National University of Singapore, Singapore, Singapore; ^2^School of Pharmacy, Monash University Malaysia, Bandar Sunway, Malaysia

**Keywords:** CNS trauma, secondary injuries, neuronal regeneration, cell penetrating proteins, biopolymers, controlled drug release

## Abstract

Traumatic brain injury (TBI) remains one of the leading causes of morbidity and mortality amongst civilians and military personnel globally. Despite advances in our knowledge of the complex pathophysiology of TBI, the underlying mechanisms are yet to be fully elucidated. While initial brain insult involves acute and irreversible primary damage to the parenchyma, the ensuing secondary brain injuries often progress slowly over months to years, hence providing a window for therapeutic interventions. To date, hallmark events during delayed secondary CNS damage include Wallerian degeneration of axons, mitochondrial dysfunction, excitotoxicity, oxidative stress and apoptotic cell death of neurons and glia. Extensive research has been directed to the identification of druggable targets associated with these processes. Furthermore, tremendous effort has been put forth to improve the bioavailability of therapeutics to CNS by devising strategies for efficient, specific and controlled delivery of bioactive agents to cellular targets. Here, we give an overview of the pathophysiology of TBI and the underlying molecular mechanisms, followed by an update on novel therapeutic targets and agents. Recent development of various approaches of drug delivery to the CNS is also discussed.

## Introduction

Traumatic brain injury (TBI) has been one of the leading causes of morbidity, disability and mortality across all ages (Bruns and Hauser, 2003; Dewan et al., 2018). Globally, more than 50 million individuals suffer from TBIs each year (Maas et al., 2017). As of 2005, approximately 3.17 million TBI survivors experience post-traumatic complications ranging from neurological, psychosocial problems to long-term disability (Zaloshnja et al., 2008; Bazarian et al., 2009). The immense expenditure on clinical management of TBI patients and associated socioeconomic problems have imposed a heavy burden on the healthcare system and the society (Finkelstein et al., 2006). While increasing understanding of the clinical characteristics and the underlying complex pathophysiological mechanisms of TBI has led to the development of novel and promising therapeutic approaches that show promising effects in preclinical studies and phase I/II trials, majority of them turn out to be unsuccessful in phase III clinical trials. In fact, more than 30 clinical trials of TBI pharmaceutical agents for diagnostics or therapeutic purposes have failed over the past three decades. This review presents an overview of the molecular and cellular events in the pathogenesis of TBI. An update on potential druggable targets and new direction of treatment is provided, followed by a discussion on various approaches to delivering these therapeutics in a controlled manner.

## Categories of TBI

According to the unique physical mechanisms of insult, TBI can be divided into three categories: (i) closed head; (ii) penetrating; and (iii) explosive blast TBI. Clinical features of TBI include prolonged coma, headache, nausea, aphasia, seizures, amnesia and behavioral abnormalities such as aggression and anxiety, which occur within seconds to minutes after TBI; however, some of these manifestations can persist up to months and years (Bruns and Hauser, 2003; Andriessen et al., 2010).

Closed head TBI is typically caused by blunt impact incurred mainly from motor vehicle accidents, falls and sports activities. The incidence rate of this form of TBI is the highest amongst the civilian population. The strong blunt and compression contact force disrupts normal functioning of the brain directly underneath the site of impact, thus causing immediate damage to brain vasculature and neuronal cells. Brain displacement due to vibrations and shocks generated during the impact can also lead to compression of brain tissues and reduction of cerebral blood flow. Both mechanisms eventually result in focal localized contusions or diffuse injury to other brain regions.

Penetrating TBI results when a foreign body penetrates the skull and traverses through the dura into brain parenchyma. Similar to closed head TBI, laceration of brain tissues primarily causes focal damages, intracranial hemorrhage, cerebral edema and ischemia. The invasion of fast-moving projectile can lead to tissue cavitation, which further exacerbates injuries. The type and severity of neurological damage are dependent on the size, speed, route and strength of the external body penetrating the brain. Due to exposure of brain tissue to the harsh environment, the chance of infection is relatively high in this form of TBI. With the invasive nature of this type of injury, penetrating TBI is associated with acute medical complications such as respiratory failure, pneumonitis, hypotonia and cerebrospinal leakage in comparison to closed head TBI (Black et al., 2002).

With the high prevalence of casualties suffering from war-related TBI in the 20th century mainly in Afghanistan and Iraq, explosive blast TBI has recently been considered as a new category (Warden, 2006). Unlike closed head and penetrating TBI, the brain is compromised by rapid pressure shock waves generated from explosion, which transmits a tremendous amount of energy from the skull into the enclosed brain parenchyma (Ling and Ecklund, 2011). The effects of blast injury can be divided into different patterns: primary (shock wave causing internal damage), secondary (penetrating), tertiary (physical injury by blast wave) and quaternary (other than the first three classes) depending on the injury outcome at different stages of blast-induced injury (Cernak and Noble-Haeusslein, 2009; Risdall and Menon, 2011). Kinetic energy generated in the blast causes deformation of the brain, thus creating a widespread diffuse injury in both the gray and the white matter, leading to neuronal cell death, axonal injury, compromised blood-brain-barrier (BBB), vasospasm, pseudoaneurysm formation, hyperemia, contusion and cerebral edema (Cernak and Noble-Haeusslein, 2009). Apart from the clinical characteristics mentioned above, post-traumatic stress disorder is frequently associated with explosive blast TBI, and research has shown a high occurrence rate in TBI survivors (Risdall and Menon, 2011).

## Pathophysiology of TBI

Damages of neuronal tissues associated with TBI fall into two categories: (i) primary injury, which is directly caused by mechanical forces during the initial insult; and (ii) secondary injury, which refers to further tissue and cellular damages following primary insult.

### Primary Brain Injuries

The immediate impact of different mechanical insults to the brain can cause two types of primary injuries: focal and diffuse brain injuries. Studies have demonstrated that the co-existence of both types of injuries is common in patients who suffered from moderate to severe TBI (Skandsen et al., 2010); however, diffuse axonal injury (DAI) accounts for approximately 70% of TBI cases. As a consequence of lacerations, compression and concussion forces, closed head TBI and penetrating TBI exhibit focal brain damage with evidence of skull fracture and localized contusion at the core of injury site (coup; Schmidt et al., 2004). Necrotic area of neuronal and glial cells is concentrated at the coup with compromised blood supply, causing the occurrence of hematoma, epidural, subdural and intracerebral hemorrhages at confined layers of the brain. Secondary contusion may develop in tissues opposite to or surrounding the coup (contre-coup) due to secondary impact when the brain rebounds and strikes the skull (Schmidt et al., 2004). Depending on the severity of the injury, it can lead to cognitive deficits, behavioral changes and hemiparesis. In contrast to focal injury, the main mechanism of diffuse brain injury is non-contact forces of rapid deceleration and acceleration which cause shearing and stretching injury in cerebral brain tissues. The strong tensile forces damage neuronal axons, oligodendrocytes and blood vasculature, leading to brain edema and ischemic brain damage (Smith et al., 2003). The hallmark feature of diffuse TBI is extensive damage of axons predominantly in subcortical and deep white matter tissue such as the brain stem and corpus callosum, which involves impairment of axonal transport and degradation of axonal cytoskeleton. Notably, these axonal damages can persist up to months following TBI, suggesting an association with delayed secondary pathology of hemorrhages and brain edema (Saatman et al., 2008). The degree of axonal injury and neuronal degeneration determines the severity of TBI. Interestingly, while explosive blast TBI is a result of shock waves instead of inertial forces, it displays the characteristics of a typical diffuse brain injury.

### Secondary Brain Injuries

**Figure 1:**
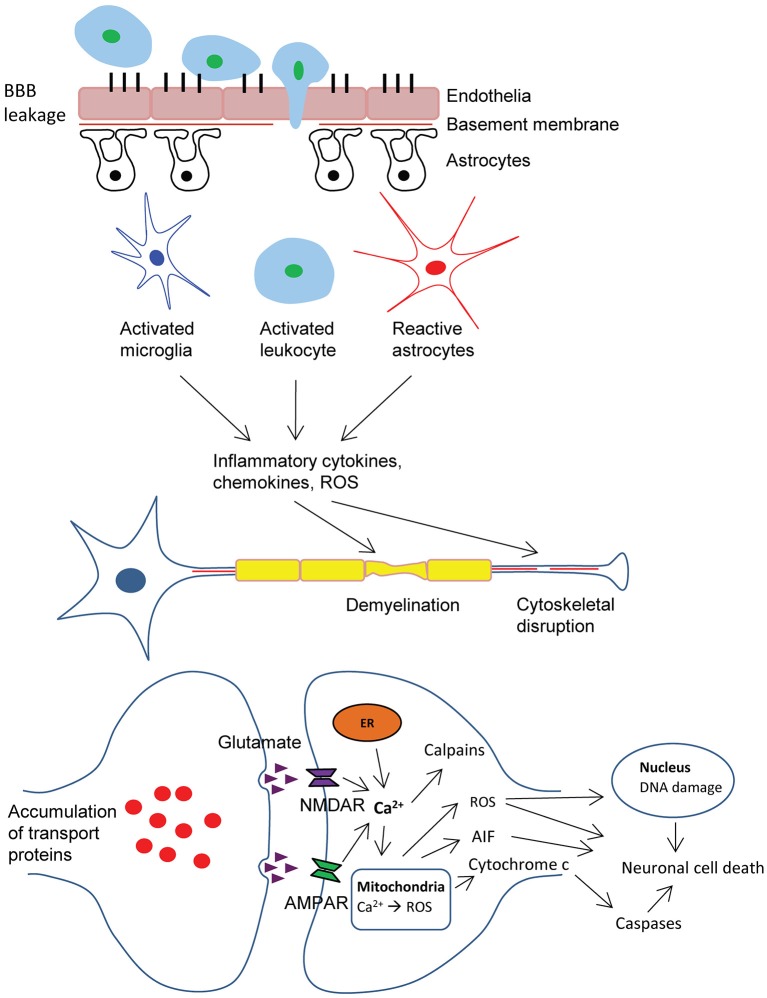
Schematic representation of pathophysiology of traumatic brain injury (TBI). BBB dysfunction caused by TBI insult allows transmigration of activated leukocytes into the injured brain parenchyma, which is facilitated by an upregulation of cell adhesion molecules. Activated leukocytes, microglia and astrocytes produce ROS and inflammatory molecules such as cytokines and chemokines that contribute to demyelination and disruption of axonal cytoskeleton, leading to axonal swelling and accumulation of transport proteins at the terminals, hence compromising neuronal activity. Progressive axonal damage results in neurodegeneration. In addition, astrogliosis at the lesion site causes glial scar formation, which creates a non-permissive environment that impedes axonal regeneration. On the other hand, excessive accumulation of glutamate and aspartate neurotransmitters in the synaptic space due to spillage from severed neurons, glutamate-induced aggravated release from pre-synaptic nerve terminals and impaired reuptake mechanisms in traumatic and ischemic brain activate NMDA and AMDA receptors located on post-synaptic membranes, which allow the influx of calcium ions. Together with the release of Ca^2+^ ions from intracellular store (ER), these events lead to the production of ROS and activation of calpains. As a result of mitochondrial dysfunction, molecules such as apoptosis-inducing factor (AIF) and cytochrome c are released into the cytosol. These cellular and molecular events including the interaction of Fas-Fas ligand ultimately lead to caspase-dependent and -independent neuronal cell death. BBB, blood-brain-barrier; ROS, reactive oxygen species; AMPA, α-amino-3-hydroxy-5-methyl-4-isoxazolepropionic acid; NMDA, N-methyl-d-aspartate; ER, endoplasmic reticulum.

The biochemical, cellular and physiological events that occur during primary injury often progress into delayed and prolonged secondary damages which can last from hours to years. Mechanistically, a number of factors contribute to secondary injuries, which include excitotoxicity, mitochondrial dysfunction, oxidative stress, lipid peroxidation, neuroinflammation, axon degeneration and apoptotic cell death (Ray et al., 2002; Figure 1).

#### Excitotoxicity

Studies in both animals and humans have demonstrated that BBB breakdown and primary neuronal cell death during TBI induce excessive release of excitatory amino acids such as glutamate and aspartate from presynaptic nerve terminals (Faden et al., 1989; Chamoun et al., 2010). The presence of excessive glutamate during TBI is also contributed by a failure of glutamate re-uptake due to the dysfunction of glutamate transporters. There has been evidence that shows a 40% decline in the expression of astrocytic sodium-dependent glutamate transporters GLAST (EAAT1) and GLT-1 (EAAT2) within 24 h following TBI, leading to a significant decrease in the resorption of glutamate (Rao et al., 1998; van Landeghem et al., 2006). These excitatory amino acids activate both ionotropic glutamate receptors (iGluRs) and metabotropic glutamate receptors (mGluRs). Members of iGluRs such as N-methyl-d-aspartate (NMDA) receptor and α-amino-3-hydroxy-5-methyl-4-isoxazole propionate (AMPA) receptor are ligand-gated ion channels that allow Na^+^, K^+^ and Ca^2+^ ionic flux upon binding to glutamate, causing membrane depolarization in neurons (Meldrum, 2000). NMDA receptor is peculiar in that it is also voltage-gated and is permeable to Ca^2+^ ions. Hyperactivation of AMPA and NMDA receptors by excessive glutamate has been shown to alter ion homeostasis in postsynaptic neurons by allowing influx of extracellular Ca^2+^ and Na^+^ ions (Sun et al., 2008; Brustovetsky et al., 2010). NMDA-induced surge in intracellular Ca^2+^ initiates the activation of various downstream signaling molecules, including Ca^2+^/calmodulin-dependent protein kinase II (Folkerts et al., 2007), mitogen activated protein kinases (MAPK; Lu et al., 2008) and protein phosphatases (Bales et al., 2009). Protein kinase C is also activated to couple to NMDA receptors, thereby enhancing Ca^2+^ influx into postsynaptic neurons (Luo et al., 2011). Similarly, activation of AMPA receptors can also trigger the MAPK pathway through calcium-dependent mechanisms (Schenk et al., 2005). Activation of NMDA receptors by glutamate promotes the production of reactive oxygen species (ROS; Reynolds and Hastings, 1995; Girouard et al., 2009) and nitric oxide (NO; Sattler et al., 1999), which further exacerbates secondary cell injury. Unlike iGluRs, mGluRs regulate Ca^2+^ and downstream signaling *via* GTP-binding proteins. Glutamate stimulation of mGluRs triggers the activation of phospholipase C/inositol-1,4,5-triphosphate, which in turn mobilizes Ca^2+^ release from intracellular stores into the cytosol and triggers the signaling cascades in injured CNS (Weber, 2012). Excessive Ca^2+^ in the cytosol also activates a number of proteins that cause apoptotic cell death, such as calcineurin, calpain and caspases. In addition, accumulation of Ca^2+^ and ROS leads to impairment of mitochondrial function, further aggravating the deregulation of Ca^2+^ and ROS homeostasis. In summary, excessive stimulation of glutamate receptors due to massive release of excitatory neurotransmitters leads to post-traumatic oxidative stress and excitotoxic cell death over an extended period, which correlate with increased mortality rate and worsened 6-month neurological outcome (Deshpande et al., 2008; Chamoun et al., 2010).

#### Mitochondrial Dysfunction

Mitochondrial dysfunction is one of the hallmark events of TBI (Xiong et al., 1997), which contributes to metabolic and physiologic deregulations that cause cell death. The sequestration of intracellular Ca^2+^ and influx of excessive ions into mitochondria results in the production of ROS, depolarization of mitochondrial membrane and inhibition of ATP synthesis (Lifshitz et al., 2004; Singh et al., 2006). This leads to the breakdown of electron transport chain and impairment of oxidative phosphorylation processes, thus disrupting the restoration of metabolic reactions for cell survival and regulation of calcium cycle. Mitochondrial permeability transition pore (mPTP) is also activated under these conditions. Conformational change of an inner membrane protein adenine nucleotide translocator (ANT) upon binding to cyclophilin D leads to the opening of mPTP and an increase in inner membrane permeability (Susin et al., 1998; Naga et al., 2007; Tsujimoto and Shimizu, 2007), further contributing to mitochondrial pathology. Electron microscopy analysis of mitochondria has revealed significant swelling and structural damages such as disruption of cristae membrane and loss of membrane potential. Furthermore, mitochondrial proteins such as cytochrome c and apoptosis-inducing factor (AIF) which play crucial roles in apoptotic cell death are released into the cytosol (Sullivan et al., 2002; Singh et al., 2006).

#### Release of Reactive Oxygen Species and Lipid Peroxidation

Accumulating evidence suggests that oxidative stress contributes to TBI pathogenesis to a significant extent. Endogenous ROS and free radicals are constantly generated following TBI from various sources, like enzymatic processes, activated neutrophils, excitotoxic pathways and dysfunctional mitochondria (Xiong et al., 1997; Shohami and Kohen, 2011). On the other hand, the accumulation of Ca^2+^ after TBI increases the activity of nitric oxide synthases (NOS), which aids in the production of NO. The reaction between excessive NO and free radical superoxides results in the formation of peroxynitrite (PN), which induces oxidative damage and can be measured by detecting oxidative markers such as 3-nitrotyrosine (3-NT) and 4-hydroxynonenal (4-HNE; Hall et al., 2004). *In vivo* studies have shown an increase in the levels of 3-NT and 4-HNE in ipsilateral cortex and hippocampus (Hall et al., 2004; Singh et al., 2006; Deng et al., 2007; Ansari et al., 2008a) after TBI. Oxidative stress is also associated with impaired synaptic plasticity in injured cortex and hippocampus, with concomitant loss of the synaptic proteins synapsin-1 and PSD-95 from 24 to 48 h post-injury (Ansari et al., 2008a,b). These ROS react not only with proteins and DNA but also polyunsaturated fatty acids in membrane phospholipids which in turn form lipoperoxyl radicals, further damaging cell membranes. The increase in permeability of mitochondria membrane and the oxidation of membrane proteins leads to an alteration of ion transport. Abnormal Ca^2+^ accumulation, for instance, has profound implications in prolonged excitotoxicity (Praticò et al., 2002). In short, the persistent release of highly reactive oxygen free radicals and the associated elevation in the level of ROS-mediated lipid peroxidation in TBI impose adverse effects in brain plasticity, cerebral blood flow, and promote immunosuppression (Ansari et al., 2008a).

#### Neuroinflammation

Within the acute post-TBI period of 24 h, dysfunction of BBB allows infiltration of circulating neutrophils, monocytes and lymphocytes into the injured brain parenchyma (Lotocki et al., 2009). Analysis of cerebrospinal fluid (CSF) and post-mortem tissue of TBI patients (Buttram et al., 2007; Frugier et al., 2009; Goodman et al., 2009) and tissue of TBI rodents (Ahn et al., 2004; Lotocki et al., 2009; Semple et al., 2010) revealed that these polymononuclear leukocytes release complement factors and pro-inflammatory cytokines such as IL-1β, IL-6 and TNF-α, as evident by an increase in the corresponding mRNA and protein 24 h post-trauma. Sustained upregulation of various cytokines was found to be associated with altered BBB permeability, formation of edema and neurological deficits. As a member of the Fas superfamily, TNF-α interacts closely with Fas ligand which in turn activates caspases that are essential for programmed cell death (Morganti-Kossmann et al., 2002). Chemokines such as MIP-α, MCP-1 and IL-8 (CXCL8) are significantly upregulated post-trauma, which act synergistically and are involved in further recruitment of leukocytes to the injury site (Kossmann et al., 1997; Buttram et al., 2007; Bye et al., 2007; Semple et al., 2010). Furthermore, upregulated expression of ICAM-1 and VCAM-1, which are ligands for endothelial and leukocyte cell adhesion receptors facilitates the interaction of leukocytes and immune cells with endothelium, hence promoting their recruitment to the injured site (Carlos et al., 1997; Rancan et al., 2001). Prolonged and delayed neuroinflammation in turn recruits macrophages, activates resident microglia cells and promotes astrogliosis (Morganti-Kossmann et al., 2007; Bye et al., 2011). Progressive phagocytosis and persistent inflammatory responses are evident by the accumulation of macrophages and activated microglia in TBI survivors years after injury (Gentleman et al., 2004; Johnson et al., 2013).

#### Axonal Degeneration

Wallerian degeneration is widely observed within minutes after DAI. Immediate mechanical damage leads to disorganization of axonal cytoskeletal network, which consists of longitudinally oriented neurofilaments and microtubules (Tang-Schomer et al., 2010). Together with constant calcium-mediated proteolysis, acute axonal damage can progress and develop into delayed and secondary axotomy days and months following the initial trauma, which is characterized by degradation of myelin sheath, impairment of axonal transport and accumulation of axonal transport proteins (Povlishock, 1992; Saatman et al., 2003; Büki and Povlishock, 2006). Formation of retraction bulbs due to disassociation of axonal connections and accumulation of axonal transport proteins in the node can eventually result in prolonged swelling of injured axons and apoptotic cell death of neurons and oligodendrocytes (Büki and Povlishock, 2006). As the hallmark of DAI, these retraction bulbs can be detected by the axonal markers β-amyloid precursor protein (β-APP) and neurofilament (NF) as early as 1 day post-TBI and up to 2 weeks in experimental models of diffuse TBI. Retraction bulbs are predominantly found in corpus callosum and pyramidal tracts of brain stem (Pierce et al., 1996; Hellewell et al., 2010), though their presence in hippocampus, cortex, cingulum, the internal and external capsule has also been reported (Hellewell et al., 2010). Hellewell et al. (2010) has demonstrated the association between axonal damage in corpus callosum and infiltration of neuroinflammatory cells (microglia and macrophages) which would lead to disruption of blood vasculature, degradation of axons, damage of oligodendrocytes and deformation of white matter.

#### Glial Scar and Myelin-Associated Axonal Growth Inhibitors

Insults to the CNS often trigger activation and proliferation of astrocytes. The resulting reactive astrocytes infiltrate into the lesion site and undergo reactive astrogliosis, which involves hypertrophy and an increase in the complexity of their processes. Intermingle of astrocytic processes with oligodendrocytes, meningeal cells, microglia and fibroblasts gradually develop into a scar-like structure, which has long been implicated as a major physical impediment to axonal regeneration and counteracts TBI recovery (Fawcett and Asher, 1999). Recent findings however suggest that chondroitin sulfate proteoglycans (CSPGs) such as neurocan and versican in glial scar, which are upregulated following CNS injury, are in fact the molecular barrier that impedes axonal regeneration (Asher et al., 2000, 2001, 2002). Together with other inhibitory molecules in glial scar, such as tenascins and semaphorin 3A, these molecules constitute a non-permissive milieu for axonal growth (Zhang et al., 1997; Pasterkamp et al., 2001; De Winter et al., 2002). Interestingly, RhoA pathway is implicated in mediating their inhibitory effects because blockade of RhoA activity or its downstream effectors promotes permissive growth of neuronal axon on these substrates (Winton et al., 2002; Monnier et al., 2003). The signaling cascades triggered by semaphorin 3A in glial scar, for instance, involve neuropilin-plexin receptor complex and the activation of Rho GTPases, which are believed to induce growth cone collapse through the regulation of F-actin cytoskeleton (Pasterkamp and Kolodkin, 2003).

In addition, damaged myelin in severed axon causes the exposure of axon outgrowth inhibitors such as myelin-associated glycoprotein (MAG), oligodendrocyte myelin glycoprotein (OMgp) and Nogo-A (Chaudhry and Filbin, 2006). Intriguingly, these myelin-associated inhibitors bind specifically to Nogo receptor (NgR) complex on neuronal membrane, which consists of the co-receptors p75^NTR^, Troy and LINGO-1 (Wang et al., 2002; Mi et al., 2004; Park et al., 2005). These inhibitors trigger the activation of RhoA GTPases and Rho kinase that can induce growth cone collapse and retraction of neurites (Nash et al., 2009). In fact, post-mortem analysis of traumatized human brain tissues revealed an increase in the expression of RhoA and RhoB proteins in reactive glia and swollen neurites, which could persist up to months after TBI (Brabeck et al., 2004). In experimentally-induced focal brain injury, active RhoA was found to be accumulated at the lesioned cortex and hippocampus 18 h post-trauma (Dubreuil et al., 2006; Zhang Z. et al., 2008). While the precise role of Rho GTPase pathway in TBI requires further investigation, its involvement in related forms of CNS injuries like spinal cord injury and cerebral ischemia has been well established (Dubreuil et al., 2003; Yagita et al., 2007). It is suggested that RhoA not only inhibits axonal regeneration but also plays a role in apoptotic responses after TBI as constant upregulation of active RhoA impairs regeneration of axons and neurites.

#### Apoptotic Cell Death

Apoptotic cell death of neurons and oligodendrocytes are hallmarks of secondary brain injury (Beer et al., 2000; Grady et al., 2003). Smith et al. (1997) have reported that neuronal cell death is evident in human hippocampus for up to 1 year after TBI. These apoptotic events involve the activation of cysteine proteases such as caspases and calpain, and can be triggered by the interaction of various neurochemical, cellular and molecular pathways such as extracellular signal-regulated kinase (ERK), p38 MAPK, janus kinase/signal transducer and activator of transcription (JAK/STAT; Kawasaki et al., 1997; Mori et al., 2002; Raghupathi, 2004; Zhao et al., 2011). Apoptotic cell death caused by caspase-dependent mechanisms can be induced by the extrinsic death receptor pathway or the intrinsic mitochondrial pathway (Stoica and Faden, 2010). Extrinsic pathway involves the interaction of TNF and Fas with their specific receptors on cell surface, whereas intrinsic pathway is activated when cytochrome c is released after mitochondrial depolarization (Sullivan et al., 2002). Cytochrome c forms an ATP-dependent complex with apoptotic activating protein-1 and ATP in the cytosol. Both mechanisms activate the caspase-dependent downstream signaling through upregulation and activation of caspase 8 and 9 which ultimately lead to the cleavage and activation of caspase 3 (Clark et al., 1999, 2000; Zhang et al., 2003). On the other hand, caspase-independent apoptosis in TBI can be initiated by the activation of calpains through proteolysis of cytoskeletal proteins such as spectrin and NF proteins (Deng et al., 2007) and the release of mitochondrial proteins such as AIF (Hong et al., 2004), Smac/DIABLO, Omi/HtrA_2_, poly (ADP-ribose) polymerase-1 and endonuclease G (Mammis et al., 2009). These mitochondrial proteins translocate into the nucleus and activate downstream signaling molecules, resulting in DNA damage and chromatin condensation in neuronal and glial cells. Apoptosis can be regulated by anti-apoptotic proteins such as the Bcl-2 family and death-inducing factors such as Bax (Wennersten et al., 2003). Studies have shown that Bcl-2 protein expression is significantly upregulated in brain tissues of TBI patients (Clark et al., 1999). Similarly, a 25% increase in Bax protein was observed in traumatic rat brain (Raghupathi et al., 2003).

#### Impairment of Autophagy and Lysosomal Pathways

Autophagy is an adaptive homeostatic process that regulates the turnover of cellular organelles and proteins through lysosome-dependent degradation pathway (Mizushima et al., 2008). Autophagy plays an important role in cytoprotection, maintenance of cell stability and survival through elimination of abnormal intracellular proteins or organelles when cells are severed or under stress, though it is also implicated in the regulation of apoptotic cell death, inflammation, and adaptive immune responses (Maiuri et al., 2007). Macroautophagy is amongst the best-characterized autophagy subtype, which is a multi-step process that involves sequestration of cytoplasmic components such as damaged organelles and proteins in double-membrane structures known as autophagosomes, followed by fusion with lysosomes whereby proteolytic degradation occurs (Mizushima, 2007). This autophagic flux is under tight regulation by members of the autophagy-related (ATG) protein family such as ATG9, the autophagosome marker protein LC3-II that is involved in the recruitment of substrates for autophagic degradation, and the beclin 1 protein which is essential for autophagosome formation. Accumulating evidence suggests the involvement of autophagy-lysosome pathway in secondary injury processes of TBI and SCI, though whether it plays beneficial or detrimental roles remains controversial. Upregulation of autophagic markers and accumulation of autophagosomes have been observed in early phase of secondary injury, which correlate with severity and can persist for weeks to months (Diskin et al., 2005; Clark et al., 2008; Sakai et al., 2014; Au et al., 2017). The increase in autophagic flux, which can be potentiated by rapamycin is associated with improved neurobehavioral function, enhanced neuronal survival, reduced inflammation and gliosis in injured brain (Erlich et al., 2007; Zhang Y. B. et al., 2008). In fact, many neuroprotective drugs alleviate TBI-induced secondary injury by activating autophagy (Ding et al., 2015; Gao et al., 2017; Zhang et al., 2017). Nonetheless, lysosomal function is often found to be compromised in TBI, which involves an increase in lysosomal membrane permeability. This leads to an impairment of autophagic flux and pathological accumulation of autophagosomes and their cargo, causing neuronal cell death and exacerbating the severity of trauma (Sarkar et al., 2014).

## Potential Therapeutics

Since primary injuries in TBI usually involve acute physical damages and necrotic cell death that are unlikely to be reversible, treatment regimens mainly aim to stabilize the site of injury and prevent it from secondary damage. As mentioned above, secondary injuries are caused by an array of risk factors and develop in a progressive manner. This provides a window for therapeutic intervention of events that could induce further loss of neurons and glial cells beyond the injury epicenter, which include persistent inflammatory response, excitotoxicity, oxidative stress and apoptotic cell death (Ray et al., 2002). Extensive research has been dedicated to gain a better understanding of the underlying mechanisms of secondary brain injuries (Table 1), in the hope of developing more effective therapeutic strategies to target multiple stages.

**Table 1:** Summary of the pathophysiology, therapeutic targets and potential therapies in traumatic brain injuries.

Pathophysiology	Therapeutic targets	Potential therapies	Clinical trials	Treatment efficacy
Excitotoxicity	Glutamate receptors, Ca^2+^ channels, calpains/caspases	Glutamate receptor antagonists HU211 (Dexanbionol; Nadler et al., 1993, 1995; Shohami et al., 1995), MK 801 (Goda et al., 2002; Imer et al., 2009), NBQX (Follett et al., 2000)	Dexanbionol: NCT00129857	Neuroprotective effect in experimental TBI but not efficacious in clinical trials (Maas et al., 2006)
		Ca^2+^ channel inhibitors (S)-emopamil (Okiyama et al., 1992, 1994), SNX-111 (Ziconotide; Samii et al., 1999) and SNX-185 (Lee et al., 2004; Shahlaie et al., 2009), Nimodipine (Veng et al., 2003), Nicarpine (Compton et al., 1990)		
		Calpain/caspase inhibitors MDL 28170 (Kawamura et al., 2005), Z DEVD-fmk (Knoblach et al., 2004)		
Mitochondrial dysfunction	ROS, mPTP components, cytochrome c	Neuroprotectants Cyclosporine A (Okonkwo and Povlishock, 1999; Sullivan et al., 1999)	NeuroSTAT: NCT01825044; EudraCT 2012-000756-34	Anti-oxidative effect reduces axonal damage and mitochondrial dysfunction in animal TBI. Phase IIa trial confirmed drug safety and BBB permeability (Kelsen et al., 2019)
Oxidative stress	ROS	Anti-inflammatory agents Methylprednisolone (Hall, 1992) Neuroprotectants Cyclosporine A (Turkoglu et al., 2010)	Methylprednisolone: ISRCTN74459797; NCT00004759	Anti-inflammatory and anti-oxidative effects. Early administration of methyl-prednisolone is associated with higher risk of death in patients with head injury (Thompson and Bakshi, 2005)
Neuroinflammation	Pro-inflammatory chemokines, complement factors	Anti-inflammatory agents Minocycline (Tikka and Koistinaho, 2001; Bye et al., 2007; Filipovic and Zecevic, 2008; Ng et al., 2012)	Minocycline: NCT01058395; NCT02802631	Anti-inflammatory and anti-apoptotic effects. Erythropoietin shows no beneficial effect in moderate or severe TBI patients (Nichol et al., 2015)
		Anti-apoptosis Erythropoietin (Yatsiv et al., 2005; Chen et al., 2007)	Erythropoietin: NCT00987454; NCT00313716	
Axonal degeneration	Calpains, NOS	Glutamate receptor antagonists NBQX (Follett et al., 2000; Goda et al., 2002)		Anti-apoptotic, anti-inflammatory, neuroprotection
		Calpain inhibitors MDL 28170 (Buki et al., 2003; Ai et al., 2007; Czeiter et al., 2009)		
		Anti-inflammatory agents Minocycline (Siopi et al., 2011)		
		Neuroprotectants Cyclosporine A (Okonkwo and Povlishock, 1999; Okonkwo et al., 1999)		
		Anti-apoptosis Erythropoietin (Yatsiv et al., 2005)		
		Stem cells therapy Marrow stromal cells (Mahmood et al., 2004b), mesenchymal stem cells (Kim et al., 2009), fetal stem cells (Riess et al., 2002; Skardelly et al., 2011)		
		Neurotrophic factors BDNF, NGF (Kromer, 1987; Dixon et al., 1997; Sinson et al., 1997), bFGF (Dietrich et al., 1996), EGF (Laskowski et al., 2005)		
Apoptosis	Caspases, calpains, cytochrome c	Calpain/caspase inhibitors MDL 28170 (Kawamura et al., 2005; Thompson et al., 2010), Z DEVD-fmk (Clark et al., 2000; Knoblach et al., 2004)		Anti-apoptosis
		Anti-apoptosis Erythropoietin (Yatsiv et al., 2005; Liao et al., 2008)		
		Stem cells therapy Mesenchymal stem cells (Kim et al., 2009)		
Impaired autophagy-lysosomal pathway	mTOR	Rapamycin (Erlich et al., 2007; Zhang Y. B. et al., 2008), Luteolin (Xu et al., 2014)		Neuroprotection
Myelin-derived inhibitors	Nogo and NgR, MAG, OMgp, RhoA	Myelin inhibitors IN-1 antibody against Nogo-A (Yu et al., 2008), DNA vaccine against myelin inhibitors (Zhang et al., 2009)	IN-1 antibody: NCT03935321	Intrathecal administration of anti-Nogo-A to SCI patients is well-tolerated in phase I trial (Kucher et al., 2018)
		RhoA inhibitors C3 transferase (Tan et al., 2007; Höltje et al., 2009; Boato et al., 2010)	Cethrin (BA-210: NCT00500812; VX-210: NCT02669849)	Treatment of SCI patients with Cethrin is well-tolerated in phase I/IIa trial (McKerracher and Anderson, 2013)
Glial scar	CSPGs, tenascins, semaphorins	Glial scar Chondrotinase ABC (Bradbury et al., 2002; Barritt et al., 2006; Lin et al., 2008)		Chondrotinase ABC promotes axon outgrowth and regeneration in SCI animals
		RhoA inhibitor C3 transferase (Monnier et al., 2003)		

### Protection of Neurons and Glia Against Excitotoxicity

#### Glutamate Receptor Antagonists

HU-211 (dexanabinol), a non-competitive NMDA receptor antagonist, has been shown to attenuate NMDA receptor-mediated neurotoxicity in neuronal cultures (Nadler et al., 1993). It is equally potent *in vivo*, as evident by a significant reduction in NMDA-induced Ca^2+^ accumulation in rat brain when administered 3 days post-trauma (Nadler et al., 1995). Post-traumatic administration of HU-211 reduces BBB dysfunction, brain edema, TNF-α production as well as apoptosis of glial and neuronal cells (Eshhar et al., 1995; Shohami et al., 1997). Similarly, another NMDA receptor antagonist MK 801 (dizocilpine) has been shown to reduce oxidative stress, microglia activation, oxidative stress, axonal damage and neuronal cell death (Goda et al., 2002; Imer et al., 2009). Importantly, these effects are associated with an improvement of cognitive function and neurological outcome (Shohami et al., 1995, 1997). Similarly, the AMPA receptor antagonist NBQX was shown to attenuate damages in neuronal axons and oligodendrocytes (Follett et al., 2000; Goda et al., 2002). While these glutamate receptor antagonists exhibit neuroprotective effects in various models of experimental TBI, they failed to improve the neurological outcome of TBI patients in clinical trials (Maas et al., 2006, 2010; Jain, 2008). The discrepancy between preclinical animal study and clinical trials in patients could have been due to the fact that glutamate-mediated excitotoxicity is an acute phenomenon shortly after primary neuronal injury. The persistent elevated level of glutamate in traumatized human brain may instead be a neuroprotective mechanism that maintains survival of spared neurons, as supported by earlier reports that demonstrated the pro-apoptotic role of NMDA-receptor antagonists in primary hippocampal neurons (Hardingham et al., 2002). In fact, NMDAR is known to mediate both neuroprotective and neurotoxic effects (Hardingham, 2009). The opposing function is believed to be due to distinct properties and differential distribution of GluN2 subunits of tetrameric NMDAR. GluN2A-containing receptors are mainly localized to synapses, while GluN2B-containing receptors are found in both synaptic and extrasynaptic locations. GluN2A is known to be pro-survival whereas GluN2B promotes cell death following excitotoxic glutamate stimulation (Liu et al., 2007). Blocking NMDAR function in a non-discriminating manner, therefore, may not reduce excitotoxicity but suppress pro-survival signals.

#### Inhibitors of Calcium Channels and Calcium-Activated Enzymes

Hyperactivation of voltage-sensitive ion channels such as L- and N- calcium channels, which causes prolonged alterations in calcium homeostasis is another important factor that contributes to excitotoxicity during secondary injuries in TBI. Many calcium channel inhibitors have in fact been demonstrated to be neuroprotective in experimental TBI. In a fluid percussion brain injury rat model, the calcium channel blocker SNX-111 (Ziconotide) was found to reduce trauma-induced calcium accumulation by 50–70% in the ipsilateral regions as early as 6 h post-trauma (Samii et al., 1999). Another calcium channel inhibitor (S)-emopamil has been shown to reduce brain edema and cerebral blood flow (Okiyama et al., 1992, 1994). Both SNX-111 and (S)-emopamil are able to ameliorate motor and cognitive deficits associated with brain injury (Okiyama et al., 1992; Berman et al., 2000; Verweij et al., 2000). With a 45% amino acid similarity, SNX-185 works in a similar mechanism as SNX-111 but with improved bioavailability and extended sustainability in the brain (Newcomb et al., 2000; Lee et al., 2004). The L-type voltage-sensitive calcium channel antagonist nimodipine was also found to have beneficial effect for memory impairment in rats, though clinical trials were terminated because of its hypotensive effects and the lack of improvement in intracranial pressure observed in TBI survivors (Bailey et al., 1991; Veng et al., 2003; Maas et al., 2010). In addition, clinical benefits are also modest in trials of the calcium channel blocker nicardipine (Compton et al., 1990). Recent studies suggested that the calpain inhibitor MDL-28170 suppresses degradation of the cytoskeletal protein α-spectrin localized at sites of neuronal damage in both TBI and hypoxic-ischemic injury, which is associated with a reduction in necrosis and apoptosis through the inhibition of calpains and caspase-3 (Kawamura et al., 2005; Thompson et al., 2010). Pre-treatment of TBI animals with MD-28170 also exerts neuroprotective effects through the preservation of axonal structure and reduction in axolemmal leakage, as demonstrated by a decrease in immunolabeling of APP (marker for defective axoplasmic transport) and RMO-14 (marker for neurofilament compaction) in injured axons (Buki et al., 2003; Ai et al., 2007; Czeiter et al., 2009). Similarly, the caspase-3 inhibitor Z-DEVD-fmk reduces neuronal cell death in neuron-glial co-culture, and is sufficient for improving neurologic function and reducing lesion volumes in induced injury in mouse and rat brain (Clark et al., 2000; Knoblach et al., 2004).

### Combating Chemical Stress to Neurons and Glia

#### Antioxidants

The immunosuppressive drug cyclosporine A, a potent regulator of mPTP, has been demonstrated to have neuroprotective effects in experimental models of TBI (Kulbe et al., 2018). Although the exact mechanistic action of cyclosporine A remains poorly understood, its administration after TBI is associated with reduced accumulation of Ca^2+^ through binding of the cytosolic phophastase calcineurin to Cyp-D at mPTP. Cyclosporine treatment also inhibits the mitochondrial release of cytochrome c and influx of Ca^2+^ into mitochondria (Sullivan et al., 2005). Furthermore, cyclosporine A exhibits anti-oxidative properties by downregulating lipid peroxidation (Turkoglu et al., 2010). These effects lead to an amelioration of axonal damage and mitochondrial dysfunction, which result in a reduction of cortical damage and an improvement in neurological outcome (Okonkwo and Povlishock, 1999; Okonkwo et al., 1999; Scheff and Sullivan, 1999; Sullivan et al., 1999, 2000, 2010; Alessandri et al., 2002; Mbye et al., 2008). Nonetheless, it should be noted that a small randomized clinical trial of cyclosporine A in TBI surprisingly showed no improvement in neurological outcome and biochemical parameters in patients as compared to healthy individuals (Mazzeo et al., 2009). Despite this, a European multi-center phase II/III clinical trial of NeuroSTAT, a drug developed by NeuroViVe in which cyclosporine A is the active ingredient, has recently been initiated in TBI patients and the outcome is yet to be evaluated.

Methylprednisolone is a synthetic glucocorticoid that has been widely used in clinical treatment of acute CNS injuries mainly because of its potency in anti-inflammation and in controlling edema in injured CNS. Interestingly, a high dose of methylprednisolone exhibits neuroprotective effects due to its anti-oxidative properties which specifically attenuates post-traumatic lipid peroxidation. Although little is known about the mechanism of the antioxidant effect of methylprednisolone, it is believed to integrate into the structure of lipid bilayer and render cell membranes more rigid, thereby limiting the mobility of lipid peroxyl radicals (Hall, 1992). Notably, methylprednisolone has to be administered at initial phase of CNS injury at an optimal concentration to ensure maximal anti-inflammatory and neuroprotective effects. Methylprednisolone was formerly incorporated into a randomized placebo-controlled trial known as CRASH in 2004. A large sample size of more than 10,000 TBI patients was recruited into the study with a 2-week follow-up period. Nonetheless, the outcome was undesirable with an increase in mortality rate (Thompson and Bakshi, 2005). In fact, rats treated with methylprednisolone also showed a significant increase in neuronal apoptosis in the hypothalamus, pituitary and hippocampus (Chen et al., 2011; Zhang et al., 2011), which are associated with memory and learning impairment (Chen et al., 2009).

#### Anti-inflammatory and Anti-apoptotic Agents

With the ability to transmigrate and diffuse across BBB, the semi-synthetic tetracycline derivative minocycline has been found to exhibit anti-inflammatory and anti-apoptotic properties in various experimental models of neurological diseases such as stroke, SCI, Alzhemier’s disease and TBI. Numerous studies have demonstrated that the neuroprotective effects of minocycline can be attributed to its inhibition of microglia activation, proliferation and production of pro-inflammatory cytokines such as IL-1β, IL-6 and TNF-α (Sanchez Mejia et al., 2001; Bye et al., 2007; Choi et al., 2007; Parachikova et al., 2010; Garrido-Mesa et al., 2013). In an experimental mouse model of closed head injury, for instance, minocycline treatment causes a marked decrease in IL-1β level in the cortex by 50%, with concomitant inhibition of microglia activation and improvement in neurological outcome (Bye et al., 2007; Ng et al., 2012). Interestingly, minocycline treatment has been found to inhibit matrix metalloproteinases and preserve BBB integrity, leading to an alleviation of cerebral edema (Homsi et al., 2009). Minocycline has also been shown to exhibit anti-apoptotic properties by inhibiting caspase activities (Sanchez Mejia et al., 2001). In addition, Siopi et al. (2011) have reported that minocycline treatment results in significant restoration of the level of neuroprotective soluble APPα 24 h post-trauma, hence contributing to the protection of damaged axons. A recent study has reported that early administration of minocycline decreases various inflammatory and glial protein markers such as MCP-1 and S100β at 51 days post-trauma, with concomitant significant improvement in locomotion, anxiety and spatial memory in an experimental rat model of mild blast TBI. This suggests that minocycline might have a long-lasting neuroprotective effect (Kovesdi et al., 2012).

Erythropoietin (EPO) belongs to type 1 cytokine superfamily. The expression of both EPO and EPO receptor is significantly upregulated in TBI, which plays an important role in neuroprotection though the exact mechanisms remain elusive (Brines et al., 2000). It is evident that the EPO/EPOR interaction allows phosphorylation of receptor-associated Jak-2, which in turn activates various signaling pathways, including caspases, Ras/MAPK, nuclear factor Kappa B and Stat-5 (Fujitani et al., 1997; Mammis et al., 2009). Intriguingly, further research indicated that EPO can exert neuroprotective effect in the absence of EPO receptor. These EPO-mediated mechanisms are found to have prominent roles in inflammatory response and apoptotic cell death (Yatsiv et al., 2005; Xiong et al., 2010). Studies in rats have demonstrated that EPO treatment suppresses neuroinflammation with evidence of significant downregulation of adhesion molecules, NF-kb and pro-inflammatory cytokines such as IL-6, IL-1β and TNF-α (Chen et al., 2007), as well as a reduction in astrocytic response and microglia activation (Yatsiv et al., 2005). EPO has also been shown to have anti-apoptotic effects by upregulation of the anti-apoptotic proteins phospho-Akt and Bcl-XL (Yatsiv et al., 2005; Liao et al., 2008). In addition, Bcl-2 gene expression is increased, with a corresponding reduction in Bax level (Liao et al., 2009). Other beneficial effects include enhanced neurogenesis, reduced production of NO, and amelioration of brain swelling, cortical tissue and axonal damage (Lu et al., 2005; Yatsiv et al., 2005; Cherian et al., 2007). These effects of EPO are associated with an improvement in cognitive and motor functions (Lu et al., 2005; Yatsiv et al., 2005; Xiong et al., 2010). In 2010, the neuroprotective effects of EPO in experimental TBI have been successfully translated into a clinical trial involving patients with moderate to severe TBI in a joint study between Australia and New Zealand. Nonetheless, the results showed that EPO did not reduce the number of patients with severe neurological dysfunction (Nichol et al., 2015).

### Promotion of Neuronal Regeneration

#### Neurotrophic Factors

Neurotrophic factors including vascular endothelial growth factor (VEGF), brain-derived neurotrophic factor (BDNF), nerve growth factor (NGF), basic fibroblast growth factor (bFGF) and epidermal growth factor (EGF) are capable of determining the post-traumatic fate of neuronal and glial cells. Administration of these growth factors following TBI can improve neurological outcome (Wu et al., 2008; Sun et al., 2009). Exogenous VEGF, for instance, increases astrocytic response, promotes angiogenesis and enhances neurogenesis in experimental model of TBI through the activation of Akt pathway and the Raf/MEK/ERK cascade (Wu et al., 2008; Thau-Zuchman et al., 2010; Lu et al., 2011). VEGF also reduces apoptotic cell death and promotes neurite outgrowth *via* Rho-dependent pathway (Jin et al., 2006).

Administration of NGF into the lateral ventricles or parenchyma of injured adult rat brain has been shown to promote survival of cholinergic septal neurons and reduce neuronal cell death, which are in accordance with the improvement in memory retention and cognitive deficits (Kromer, 1987; Dixon et al., 1997; Sinson et al., 1997). Similarly, exogenous infusion of BDNF contributes to improvement in histological deficits and neurological function, and promotion of axonal regeneration in experimental models of excitotoxicity, cerebral ischemia and SCI (Burke et al., 1994; Schäbitz et al., 1997; Namiki et al., 2000). It should be noted, however, that Blaha et al. (2000) have shown no improvement in memory loss and alterations in apoptotic cell death in both the injured cortex and hippocampus after post-traumatic intraparenchymal infusion of BDNF. In an *in vitro* model of focal trauma using rat hippocampal slice culture, bFGF and EGF treatment promotes survival of existing neurons and formation of new neurons in the dentate gyrus, as evident by NeuN immunostaining and a significant increase in BrdU-positive newborn progenitor cells, respectively (Laskowski et al., 2005). Similar beneficial effects are observed when bFGF is administered into the brain ventricles of TBI rats, which results in a significant recovery of TBI-induced neurological deficits (Sun et al., 2009).

Infusion of bFGF to rat brain 3 h after injury induced by lateral fluid percussion can still significantly reduce neuronal damage and lesion volume (Dietrich et al., 1996). In fact, severed CNS has been found to produce various growth factors after injuries. Chiaretti et al. (2008, 2009) showed a significant upregulation of NGF in the CSF of children with severe TBI, which correlates with an improvement in Glasgow recovery scores. An upregulation of BDNF and its receptor at the cortical lesion site was also observed in induced TBI in non-human primates (Nagamoto-Combs et al., 2007). Taken together, these studies suggest that neurotrophic factors are able to confer neuroprotection after TBI.

#### Suppression of RhoA GTPase

Accumulating evidence has demonstrated that central neurons have the potential to regenerate, though the process is largely suppressed by the non-permissive environment in injured CNS. Recently, the small GTPase RhoA has emerged to play a pivotal role in mediating the effect of inhibitory molecules in glial scar and damaged myelin against axonal regeneration. Exoenzyme C3 transferase is an enzyme found in *Clostridium botulinum* that ADP-ribosylates Rho proteins by transferring the ADP-ribose moiety from NAD to the acceptor amino acid residue asparagine-41 of Rho proteins, thereby blocking the downstream signaling that causes growth cone collapse and inhibition of axonal regeneration (Aktories et al., 2005). The effect of C3 transferase in promoting axonal regeneration has been extensively studied in both *in vitro* and *in vivo* animal models of SCI and peripheral nerve injury (Tan et al., 2007; Höltje et al., 2009; Boato et al., 2010; Huelsenbeck et al., 2012). Rats subjected to experimental SCI showed improvement in neurological outcomes upon treatment with C3 peptide (Boato et al., 2010). With the same enzymatic activity as the original C3 bacterial toxin exoenzyme, the C3 derivative BA-210 has been demonstrated to enhance functional regeneration in animal models of spine injuries (Lord-Fontaine et al., 2008). Importantly, it can maintain its stability after 18 months of storage at low temperatures (Lord-Fontaine et al., 2008). The drug Cethrin/VX-210 (in which BA-210 is the active ingredient) has passed phase I/IIa open-label clinical trial that assesses its safety, tolerability and treatment efficacy in SCI patients (Fehlings et al., 2011; McKerracher and Anderson, 2013), and is currently going through phase IIb/III trial to evaluate its efficacy and safety in patients with acute traumatic cervical SCI. In addition to its key roles in promoting regeneration of axons and neurites, C3 also regulates apoptosis through interaction with p53^NTR^ (Dubreuil et al., 2003). Given the wide range of cellular functions of C3 transferase in promoting CNS regeneration, combinatorial therapies of C3 transferase and other neuroprotective drugs may provide additive effect (McKerracher and Guertin, 2013). Although the significance of C3 transferase in experimental models of TBI remains to be determined, it stands to believe that the beneficial effects observed in spine injuries are also applicable to TBI given the similarities between these two forms of CNS trauma.

#### DNA Vaccine Against Myelin-Derived Axonal Growth Inhibitors

Myelin-associated axonal growth inhibitors exposed in severed axons are known to cause growth cone collapse and impede axonal regeneration. Recent studies have reported that DNA vaccines against the myelin-derived inhibitors Nogo, MAG and OMgp promote axonal repair in the corticorubral projection and improve neurological outcome in experimental models of TBI and stroke in rats (Zhu et al., 2007; Zhang et al., 2009). Immunization of rats against Nogo receptor (NgR) after induced spine injury also promotes axonal regeneration and functional recovery (Yu et al., 2007, 2008). DNA vaccination is a novel and relatively simple technique to induce an immunological response by injection of genetically engineered DNA encoding the antigen into the body so as to trigger immune system in the host. These studies demonstrated that DNA vaccine against myelin-derived inhibitors might be a promising approach to promote recovery of injured CNS. More detailed investigation is required to validate the effects and to better understand the mechanistic action and potential side effects of these DNA vaccines.

#### Surmounting Glial Scar

Recent findings suggest that glial scar not only acts as a physical barrier to impede axon regeneration, the complex cocktail of inhibitory molecules therein such as CSPGs, tenascins and semaphorins also represent a non-permissive milieu for axonal growth (Fawcett, 2006). Significant upregulation of CSPGs like neurocan, phosphacan, versican and NG2 in glial scar contributes to the failure of axon regeneration following CNS injury. Administration of the CSPG-degrading enzyme chondrotinase ABC reduces the level of CSPGs and cavitation at the lesion site within 24 h (Lin et al., 2008). *In vivo* studies of SCI have confirmed the effect of chondrotinase ABC in the promotion of sprouting and outgrowth of injured axons and the ensuing re-innervation (Bradbury et al., 2002; Yick et al., 2003; Chau et al., 2004; Barritt et al., 2006). Importantly, the improvement in axonal pathology is associated with an amelioration of neurological deficits (Bradbury et al., 2002; Barritt et al., 2006). Overexpression of chondrotinase ABC in transgenic mice has also shown regeneration of axon through astrocytic scar (Cafferty et al., 2007). The inhibitory molecules in glial scar, therefore, represent promising targets to promote regeneration in TBI.

#### Stem Cell Therapies

Loss of neurons and glia are major hallmarks in severed CNS. Replacement of these cells, therefore, represents a valid approach of therapy. Marrow stromal cells are capable of differentiating into multiple cell lineages including glia and neurons both *in vitro* and *in vivo* (Sanchez-Ramos et al., 2000; Lu et al., 2001). Rat or human bone marrow stromal cells intravenously administered into rats after TBI were found to migrate into the lesioned cortex and displayed an astrocytic and neuronal phenotype, as identified by glial (GFAP) and neuronal (NeuN) markers, respectively (Lu et al., 2001; Mahmood et al., 2004b). Marrow stromal cells also play an important role in inducing neurogenesis after TBI, as indicated by the presence of new BrdU+ proliferating cells in the contusion, subventricular zone and hippocampus (Mahmood et al., 2004b). These histological findings correlated with a sustained improvement of neurological and motor functions (Lu et al., 2001; Mahmood et al., 2004b). Similarly, mesenchymal stem cells also exhibit beneficial effects in both *in vitro* and *in vivo* TBI studies. Mesenchymal stem cells isolated from mice promote proliferation and induce GFAP expression in neural stem cell culture. Injection of mesenchymal stem cells into acute TBI model reduces the expression of various pro-inflammatory cytokines and chemokines such as IL-1β, IL-6, TNF-α, CCL2, CCL11 and CXCL (Galindo et al., 2011). In addition to anti-inflammatory effect, mesenchymal stem cells attenuate neuronal loss in the hippocampus and cortex through a reduction of caspase-3 activation and an increase in AKT activity (Kim et al., 2009). Human mesenchymal stem cells have also been shown to improve neurological function in TBI rats 2 weeks after transplantation (Kim et al., 2009).

Stem cells from human are used in many studies due to the capability to release neurotrophic factors such as NGF and BDNF, which are known for their neuroprotective effects. Transplantation of human fetal stem cells, for instance, leads to sustained improvement in motor function and memory, which is associated with a reduction in lesion volume and neuronal loss at the lesion site (Riess et al., 2002; Skardelly et al., 2011). These can also be attributed to the promotion of angiogenesis and inhibition of activated microglia post-injury (Skardelly et al., 2011). Importantly, fetal stem cells were found to differentiate into neurons and astrocytes in injured hippocampus and cortex with the release of glial-derived neurotrophic factor (Riess et al., 2002; Gao et al., 2006). A small scale phase I clinical trial on autologous marrow stromal cell transplantation in young TBI patients has shown no adverse effects though only modest neurological improvement was found (Cox et al., 2011). Tian et al. (2013) conducted a phase I/II trial in patients with sub-acute phase of TBI by intrathecal administration of autologous bone marrow-derived mononuclear cells. While no major complications were observed, improvement in function was only seen in less than half of the patients with persistent vegetative state and motor disorder (Tian et al., 2013). Expansion of this study by recruiting more subjects will provide insight into the feasibility of this approach.

#### Extracellular Vesicles and miRNAs

While stem cell therapies have demonstrated promising effects in promoting regeneration in TBI, these treatments are associated with various complications. The use of fetal embryonic stem cells undoubtedly involves legal and ethical issues. Multipotency of stem cells poses the risk of unregulated growth and tumorigenesis (Jeong et al., 2011). Administration of these cells into the body may also occlude microvasculature and trigger immune responses (Furlani et al., 2009). Besides, it is laborious to isolate, prepare and preserve viability of stem cells. As stated above, mesenchymal stem cells have recently emerged as promising candidates for TBI treatment. MSCs administered into the body were found to preferentially migrate to damaged tissue sites where they differentiate into neurons and glial cells, reducing expression of axon outgrowth inhibitory molecules, suppressing neuroinflammation and promoting the release of growth factors, with concomitant substantial improvement in neurological functions (Das et al., 2019). Interestingly, accumulating evidence suggests that the protective effect of MSCs may not be entirely due to their differentiation and replacement of severed neurons but also through the promotion of survival and proliferation of resident cells *via* paracrine release of bioactive molecules or direct cell-cell interaction (Chen et al., 2002; Mahmood et al., 2004a). In this regard, exosome released from MSCs has emerged as promising candidate that mediates these beneficial effects. Systemic administration of cell-free exosomes released by MSCs was found to promote restoration of cognitive and sensorimotor functions in rat TBI model, concomitant with neurovascular remodeling, neurogenesis in the dentate gyrus and reduced neuroinflammation (Zhang et al., 2015). Intravenous infusion of exosomes isolated from MSCs can also suppress neuroinflammation, improve cognitive and spatial learning functions in mouse after TBI (Kim et al., 2016). Exosomes are small membrane vesicles with diameter ranging from 50 to 200 nm (Trams et al., 1981; Schneider and Simons, 2013). They carry proteins, RNAs, microRNAs, lipids, and exert intercellular signaling function by transferring these cargoes to other cells *via* ligand-receptor binding and internalization (Taylor and Gercel-Taylor, 2014). For instance, exosomes released from injured sensory neurons are enriched in miR-21, a non-coding microRNA that upon phagocytosed by macrophages promotes pro-inflammatory responses. Administration of antagomir against miR-21 reduces neuropathic hypersensitivity and recruitment of inflammatory macrophages to the injury site (Simeoli et al., 2017). By contrast, miR-21 in exosomes released from neurons formerly primed by injured mouse brain extracts have recently been shown to inhibit the activity of neuronal autophagy (Li et al., 2019). Furthermore, exosomes enriched in miR-17–92 cluster have been shown to promote neurogenesis, oligodendrogenesis, and axonal outgrowth in severed CNS due to stroke (Xin et al., 2017). miR-132 carried by exosomes acts as an intercellular signal to regulate brain vascular integrity (Xu et al., 2017). In short, exosomes derived from neurons and glial cells can regulate gene expression and miRNA activities in an autocrine manner, which in general mediate neuroprotection and neurorestorative effects by promoting neurogenesis, reducing inflammation, increasing angiogenesis and tissue remodeling.

## Delivery of Therapeutic Agents to the Brain

### Overcoming Physiological Barriers

Physiological barriers such as the BBB and the blood-CSF barrier, maintained by endothelial cells separating the CNS from the peripheral circulation, are of great importance in protecting the brain. These interfaces tightly regulate the transmigration of small molecules into the CNS, hence posing challenges to drug delivery in TBI treatment. It should be noted, however, that BBB intactness is often compromised as a direct consequence of TBI. While BBB dysfunction contributes greatly to the prolonged secondary damage after TBI, it also allows therapeutic proteins or peptides administered through other entry routes such as intranasal delivery to cross the tight endothelial junctions and reach the injury site (Habgood et al., 2007; Lotocki et al., 2009; Ligade et al., 2010). In experimental TBIs, intraventricular administration of therapeutic agents is a common and feasible method to overcome these barriers by direct delivery into the CSF (Temsamani et al., 2000). In clinical management of TBI, surgical intervention is often required to relieve intracranial pressure and edema, which also provides an opportunity for direct drug delivery.

### Sustained and Controlled Drug Delivery *via* Osmotic Pumps

While the therapeutic agents discussed above demonstrate various neuroprotective effects in both *in vitro* and *in vivo* studies of TBI, the long-lasting adverse effects associated with secondary brain damage calls for the development of delivery systems that allow constant, sustained, and controlled release of these candidate therapeutics to exert their full potential in promoting recovery from TBI. In experimental models of TBI in rats, osmotic mini-pumps have been successfully used to deliver NGF and S100B neurotrophic protein into lateral ventricles in the brain at a constant rate, which results in promotion of cognitive functions (Dixon et al., 1997; Kleindienst et al., 2004). These mini-pumps are implantable and require no external power as they are driven by the pressure developed from osmotic difference between osmolytes in the pump and interstitial fluid of the body. The capability to continuously infuse drugs at a rate of microliters per hour from 1 day to a month renders osmotic mini-pump a powerful tool to evaluate the *in vivo* efficacy and toxicity of agents that have a short half-life, like proteins and peptides, though subcutaneous implantation of the pump is needed to minimize infection and allow unrestrained movement of the subject.

### Nanocarriers

In addition to osmotic pumps, encapsulation of drugs in micro- or nano- particles is emerging as promising ways to allow sustained and controlled delivery of therapeutics in TBI research. Both natural and synthetic polymers have been successfully used as drug depots, which share common features of being biocompatible, biodegradable, generally inert, as well as capable of attaching to or encapsulating small molecules and proteins (Orive et al., 2009). While biopolymer-based drug delivery systems have been applied in many tissues and organs, reports of their use in TBI treatment is limited (Heile and Brinker, 2011; Guan et al., 2013; Khalin et al., 2016). Turkoglu et al. (2010) have administered cyclosporine A-loaded natural chitosan microspheres into brain ventricles after TBI induction in rats. While it successfully reduced mitochondrial damage and lowered lipid peroxidation, the beneficial effect was, in fact, comparable to that of the control group where cyclosporine A alone was intraperitoneally injected (Turkoglu et al., 2010). This could have been due to the sub-optimal formulations of chitosan microspheres, dosage of the drug and route of administration. Other natural biopolymers commonly used for drug encapsulation include alginate and gelatin (Orive et al., 2009). One of the most popular synthetic biopolymers used as nanocarriers for drug delivery purposes is the family of poly (D,L-lactide-co-glycolide; PLGA), polylactic acid (PLA) and polyglycolic acid (PGA). Notably, these polymers are approved by the Food and Drug Administration in the US and are confirmed to be compatible with the nervous system. Depending on the application, PLGA polymers can be prepared in different dosage forms by using specific techniques (Anderson and Shive, 1997; Soppimath et al., 2001). The emulsification solvent evaporation method, for instance is widely used in fabricating PLGA microspheres (Jain, 2000). Recently, the electrospinning technique has been developed to produce nanofibers (Li et al., 2002). Both of these methodologies allow high efficiency of drug incorporation during the production process. Tan et al. (2007) have demonstrated >80% loading efficiency in the encapsulation of the recombinant protein Tat-C3 transferase, a potent RhoA inhibitor that freely enters cells, in PLGA microspheres using the water-in-oil-in-water emulsification method (Tan et al., 2007). Alternatively, drugs can be adsorbed onto pre-fabricated polymer particles.

Drug release from PLGA-based depot involves gradual degradation of the polymer when hydrogen and covalent bonds are hydrolyzed by water to form lactic and glycolic acids, which can be metabolized by Krebs cycle in the body (Park, 1995). Manipulating the ratio of lactide to glycolide monomers in the polymer allows modulation of the degradation profile, hence the rate of drug release. A higher glycolide content, for instance, correlates with faster hydrolysis and drug release. Other contributing factors include physico-chemical properties of the polymer such as solubility, porosity and molecular weight (Anderson and Shive, 1997). In addition, polymers that are end-capped with esters are more resistant to hydrolytic degradation than those with free carboxylic acid. In the *in vitro* study by Tan et al. (2007), PLGA polymers carrying uncapped (free carboxyl) and capped (lauryl ester) end groups were blended at various ratios to determine the optimal release profile for the encapsulated recombinant protein Tat-C3. Release kinetics analysis revealed that the formulation of 30% capped-70% uncapped PLGA allowed a mild initial burst while maintaining constant rate of protein release over a period of 28 days. The protein release characteristics were a result of balanced degradation rate of capped and uncapped PLGA, as well as the concomitant gradual increase in porosity of the microspheres due to formation of new internal pores within existing pores as revealed by scanning electron microscopy (Tan et al., 2007).

Since *in vivo* application of biopolymer-based drug delivery systems involves direct and prolonged contact with tissues, one of the major concerns is their biocompatibility, which can be determined according to the inflammatory responses induced after implantation into different sites of the brain, such as the striatum, lateral ventricles, frontal lobe and substantia nigra (Fournier et al., 2003; Lampe et al., 2011). While PLGA polymers are generally known to be biocompatible, some studies have reported that they induce acute inflammatory responses, as detected by immunohistochemical staining of astrocytes though it could be a non-specific consequence of mechanical trauma (Emerich et al., 1999; Lampe et al., 2011). A known issue of PLGA polymers is their adverse effects on the stability of encapsulated proteins or peptides. Loss of protein activity or integrity during the controlled released process can be attributed to protein adsorption to the polymer, or to a greater extent protein denaturation due to acidification when PLGA polymers break down to lactic and glycolic acids. The stability of encapsulated bioactive agents can be improved by incorporating pH modifiers such as calcium carbonate or magnesium hydroxide during the encapsulation process (Houchin and Topp, 2008). Similarly, proton scavengers/sponge that are basic amines, such as 1–8-bis-(dimethylamino)naphthalene can be added as excipients (Houchin et al., 2007). Furthermore, recent studies have reported inactivation of encapsulated peptides by an acylation reaction of their reactive amines with the ester bonds of PLGA (Domb et al., 1994). PEGylation of the peptide prior to encapsulation can prevent these undesirable covalent interactions with PLGA (Na and DeLuca, 2005). The resulting PEGylated peptides also exhibit reduced immunogenicity.

### Extracellular Vesicles for Drug Delivery

Exosomes are lipid bilayer membrane vesicles released by almost all cell types. Cargoes carries by exosomes are mainly molecules derived from endosomes, ranging from mRNAs, microRNAs, proteins to lipids, which vary based on cell origin (Chopp and Zhang, 2015). Recently, exosomes derived from MSCs have received attention due to their effect in promoting functional recovery in animal models of TBIs (Zhang et al., 2015). Although the underlying mechanism is not fully understood, miRNAs transferred from exosomes seemingly play a pivotal role in mediating the beneficial effect (Taylor and Gercel-Taylor, 2013). Importantly, the unique property of exosomes as natural lipid-based nanovesicles that show high biocompatibility, low immunogenicity, efficient permeability across BBB and cell membrane renders them promising candidates to be developed as novel drug delivery system for CNS (Xiong et al., 2017). Accumulating evidence suggests that exosomes transverse through membranes *via* ligand-receptor binding and internalization. Macrophage exosomes, for instance, express the integrin lymphocyte function-associated antigen 1 (LFA-1) on surface, which interacts with the highly upregulated intracellular adhesion molecule 1 (ICAM-1) on endothelial cells of BBB in inflamed brain. Intravenous administration of macrophage exosomes pre-loaded with BDNF has been shown to successfully deliver the protein to the brain (Yuan et al., 2017). Exosomes derived from choroid plexus epithelial cells express folate receptor α (FRα), which interacts with ependymal cells and mediates transverse through the CSF-brain barrier before being taken up by astrocytes and neurons in the brain (Grapp et al., 2013). These observations suggest that receptor-mediated transcytosis of exosomes can be a promising way for drug delivery to the CNS. Apart from using natural exosomes which intrinsically expressing protein or lipid ligands that bind to intended recipient cells, exosomes can also be engineered to target particular cell types or tissues by ectopic expression of specific ligands or homing peptides. Alvarez-Erviti et al. (2011) forced expressed a fusion protein between the exosomal membrane protein Lamp2b and the neuron-specific RVG peptide in exosomes isolated from dendritic cells. Purified exosomes were then loaded with siRNA directed against GAPDH and systemically introduced into mice *via* intravenous injection. Strikingly, exosome-mediated delivery of these siRNAs was found to successfully downregulate the target mRNA in neurons, microglia, and oligodendrocytes in the brain (Alvarez-Erviti et al., 2011). Since exosomes are stable and can preserve the conformation and bioactivity of proteins and nucleic acids, they serve as ideal natural vehicles for targeted drug delivery to the CNS.

### Cell Penetrating Peptides to Facilitate Cell Entry of Drugs

While the issues of sustained and controlled delivery of drugs can be resolved by various approaches described above, therapeutic agents such as peptides or proteins directed against intracellular targets often encounter difficulties in gaining access into cells because of their low membrane permeability. To improve the efficiency of cell entry, these proteins can be fused to a peculiar class of proteins known as cell penetrating proteins (CPPs), which are capable of traversing biological membranes and act as cellular delivery vehicles (Koren and Torchilin, 2012; Guidotti et al., 2017). CPPs, also commonly known as protein transduction domains, are small amphipathic peptides that contain mainly positively charged amino acids like arginine and lysine. Different unique properties and nature of these CPPs allow non-invasive internalization of conjugated peptides or small molecules through the plasma membrane (Gupta et al., 2005; Foged and Nielsen, 2008). Despite extensive characterizations of these CPPs, the exact mechanism through which they permeate the plasma membrane is still controversial and remains to be determined. Multiple mechanisms of cellular internalization have been proposed in CPPs, and the efficiency of translocation appears to be dependent on the nature of individual CPP (Koren and Torchilin, 2012). For instance, CPPs conjugated with target peptides can directly translocate across lipid bilayer through the formation of pores at the membrane. Alternatively, CPP-mediated internalization can be *via* energy-dependent endocytosis. Lastly, the CPP-cargo fusion proteins can form vesicles and inverted micelles which are capable of destabilizing cell membrane, thus releasing the conjugated proteins into cell. Specific cationic CPPs can bind to cell surface proteoglycans (heparin sulfates) for internalization of the cargo (Foged and Nielsen, 2008; Sebbage, 2009). Both *in vitro* and *in vivo* studies of CNS injuries have demonstrated successful cellular translocation of different proteins by conjugating to various CPPs, including trans-activating transcription (Tat) factor, penetratin, membrane translocating sequences, transportan and Pep-1 (Lindgren et al., 2000). Nonetheless, the concerns about cytotoxicity and specificity of these CPPs remain controversial. While majority of studies have indicated a low level of toxicity of CPPs at low concentrations, high cytotoxicity has been reported in rat neuronal cultures (Antoniou and Borsello, 2010). Further validation of the biocompatibility of CPPs is therefore required.

## Discussion

Research in traumatic injuries in the CNS has significantly expanded our understanding of the underlying pathophysiology and molecular mechanisms. While primary injuries in TBI are largely irreversible, the ensuing secondary damages that develop and progress over months to years are amenable to therapeutical interventions. Since this delayed phase of injury involves a plethora of events, which include excitotoxicity, apoptotic cell death, inhibition of axonal regeneration, neuroinflammation and oxidative stress, the devise of efficacious therapeutic strategies will need to target multiple mechanisms over an extended period. The availability of depot systems for regulated and sustained delivery of therapeutic agents that are capable of entering cells by permeating the plasma membrane will apparently allow further improvement of the bioavailability of existing drugs. More importantly, it will offer the opportunity to explore the therapeutic potential of novel agents against druggable targets. In fact, this therapeutic approach has been applied in the treatment of many neurodegenerative disorders such as Alzheimer’s disease, Huntington’s disease and Parkinson’s disease (Popovic and Brundin, 2006; Saraiva et al., 2016). While the feasibility of this strategy in the management of TBI has yet to be established, it seems promising due to the slow progression of events during secondary damages in TBI, which require continuous availability of therapeutic agents in bioactive form at non-cytotoxic concentration. TBI has become a major health and socioeconomic problem throughout the world, which imposes a significant healthcare burden to modern societies that call for more effective therapeutic means. It also represents a valid issue in defense science because of a drastic increase in subtle CNS injuries among the military when they are better protected from fatality by modern technologies.

## Author Contibutions

All authors listed have made a substantial, direct and intellectual contribution to the work, and approved it for publication.

## Conflict of Interest

The authors declare that the research was conducted in the absence of any commercial or financial relationships that could be construed as a potential conflict of interest.
